# Commentary: Differential Effects of High-Protein Diets Derived from Soy and Casein on Blood-Brain Barrier Integrity in Wild-type Mice

**DOI:** 10.3389/fnut.2018.00067

**Published:** 2018-08-08

**Authors:** Niva Shapira

**Affiliations:** ^1^Department of Nutrition, Ashkelon Academic College, Ashkelon, Israel; ^2^Rabin Medical Center, Institute of Nutrition Research, Petah Tikva, Israel

**Keywords:** dietary protein, casein, methionine, homocysteine, B-vitamins, Mediterranean diet

Following repeated observations that a high-protein diet may induce cognitive decline, and that proteins from various foods demonstrate differential effects on cognition, the recent study by Snelson et al. (1) examined potential mechanisms in a murine model. The team compared protein from different sources for influence on blood brain barrier permeability and inflammation, which are increasingly accepted as pivotal measures for central nervous system function. As casein showed much stronger effects than soy protein—having much higher methionine compared to soy protein, which is higher in cysteine—and methionine being the leading amino acid associated with elevated plasma homocysteine (Hcy) levels (2, 3), it may be assumed that high Hcy is involved in these damaging processes in the brain.

Previous studies reported that high Hcy is associated with compromised cerebrovascular function, including blood brain barrier dysfunction (4), increased permeability and neuroinflammation (5, 6), and structural and functional alterations via promotion of oxidative stress (7), with methionine being the leading amino acid associated with elevated plasma Hcy levels (2, 3).

A recent clinical study on a high-casein meal—incorporating large oral boli of 5% fat and 11% protein cottage cheese, which has mostly casein protein—showed that blood Hcy was moderately increased for about 5–8 h postprandially (8). However, this Hcy response to cheese was reduced by acute addition of vitamin B6 (pyridoxine) and folic acid. These B-vitamins, in addition to B2 (riboflavin) and B12 (cobalamin), are highly active in the methionine metabolic cycle, but largely removed with whey in the cheese production process (Figure 1) (8). Especially vulnerable is vitamin B6, a key cofactor in Hcy metabolism (9), highly dominant in the trans-sulfuration stage and critical under high-protein conditions. While the required minimum B6:protein ratio is 15–20 μg B6/g protein (10), that of cottage cheese is generally much lower, i.e., ≈1.86 μg/g (≈6.2-fold lower than the ≥11.52 μg/g of milk (11), and lower than experimental vitamin B6-depleting diet providing <0.5 μg/g (10, 12). Thus, high casein intake (including through cottage cheese) can correspondingly be perceived as a highly B6-deficient/demanding meal that if not co-supplemented, may temporarily increase Hcy.

**Figure 1:**
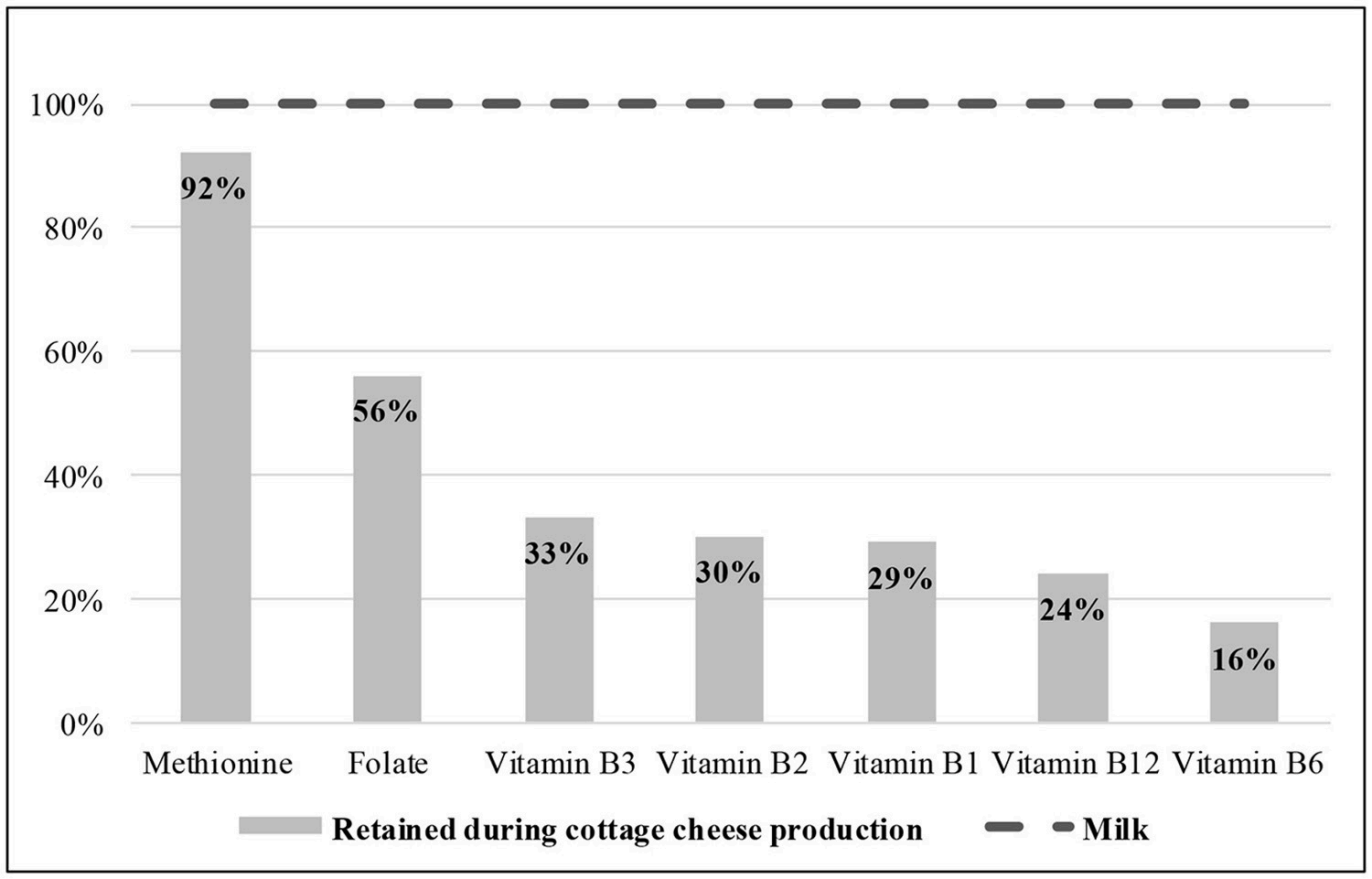
Methionine and B-vitamins, % retained in cottage cheese (USDA ID: 01015) vs. milk (representing 100%). Adapted from Shapira (8) with permission from The Royal Society of Chemistry.

Together, these studies emphasize that the potential for damage attributable to casein—through its high methionine content compared to soy protein having high cysteine content, as shown in the rat study—may be applicable to human diet, i.e., in case of high cottage cheese consumption, shown to be associated with transient (for several hours) increases in Hcy. This may suggest that high-casein foods—primarily cheese—would be better consumed together with complementary B-vitamins, preferably from foods—especially B6, e.g., from fish, poultry, sweet potato, potato, sunflower seeds, spinach, banana, and folic-acid, e.g., from beans, spinach, broccoli, leafy vegetables, tomato, bell pepper, carrots, oranges—that may compensate for the secondary B-vitamin insufficiency resulting from their removal with whey during cheese production, and would prevent temporal increase of Hcy.

With regard to the authors' aims toward dietary recommendations, we would like to emphasize the importance of food combinations, i.e., as shown by the Greek salad of the Mediterranean diet—where cheese is consumed within the salad context, together with vegetables, nuts and herbs, that would potentially compensate for insufficiencies or losses of B-vitamins, and olive oil increasing the anti-oxidative capacity vs. the Hcy-associated oxidative potential—together protecting against temporal effect of high consumption of methionine sources unopposed by sufficient B-vitamins.

Considering that cheese is currently a major protein source, including in women of childbearing age and in children—already from very young age—the findings in Snelson et al. (1) emphasize both the need to differentiate between sources of protein, especially during the critical periods of the brain development, and specifically the importance of food combinations and systematic balancing of all meals and of the complete diet.

## Author contributions

The author confirms being the sole contributor of this work and approved it for publication.

### Conflict of interest statement

The author declares that the research was conducted in the absence of any commercial or financial relationships that could be construed as a potential conflict of interest.
